# Cost-effectiveness evaluation of a radio campaign to improve full vaccination coverage among children under-five in Ethiopia

**DOI:** 10.1186/s12889-025-25441-x

**Published:** 2026-06-16

**Authors:** Bernard Appiah, Evans Otieku, Lakew Abebe Gebretsadik, Yisalemush Asefa, Martin Kampamba, Abebe Mamo, Collins Annor, Sudhakar Morankar, Kelvin Mwangilwa, Ama Pokuaa Fenny

**Affiliations:** 1 Department of Public Health, Research Program on Health Communication and Public Engagement (HCOPE), Syracuse University, Syracuse, NY United States of America; 2 Centre for Science and Health Communication, Accra, Ghana; 3 Economics Division, Institute of Statistical, Social and Economic Research (ISSER), University of Ghana, Accra, Legon, Ghana; 4 Department of Health, Behaviour & Society, Jimma University, Jimma Town, Ethiopia; 5 Department of Health Management & Policy, Jimma University, Jimma Town, Ethiopia; 6 Department of Pharmacy, University of Zambia, Lusaka, Zambia; 7 National Public Health Institute, Kabulonga, Lusaka, Zambia; 8 Department of Epidemiology and Biostatistics, School of Public Health, University of Zambia, Lusaka, Zambia

**Keywords:** Childhood vaccination, Radio campaign, Cost-effectiveness

## Abstract

**Background:**

Achieving timely childhood vaccination is a challenge in low- and middle-income countries (LMICs). A three-month intervention called 10 + 10 + 30 involved 10-minute serial radio drama on vaccination, 10-minute community health worker discussion, and a 30-minute phone-in by listeners was implemented in Jimma Zone, Ethiopia. The goal was to increase childhood vaccination and improve child health outcomes. There is limited evidence of the cost-effectiveness of a radio-only intervention on childhood vaccination in LMICs. Thus, the aim of this study was to examine the cost-effectiveness of 10 + 10 + 30 radio campaign.

**Methods:**

A scenario modeling design using evidence from a quasi-experimental trial in two districts of Jimma Zone in Ethiopia. The priority populations were all under-five children living in Ethiopia in 2021. The main outcome measures were the proportion of incremental full childhood vaccination coverage, total number of lives saved (mortality avoided), and cost per disability-adjusted life years averted.

**Results:**

From an estimated unit campaign cost of about US$0.04 per child, we projected a 134% increase in full vaccination coverage, equivalent to an additional 8 million fully vaccinated under-five children. If implemented at scale, the intervention had the potential to reduce under-five mortality by about 32%, corresponding to 288,000 lives saved and an estimated cost per averted disability-adjusted life year (DALY) of US$78.

**Conclusion:**

The study provides evidence to show that a radio vaccination campaign at scale in Ethiopia may have the potential to increase full childhood vaccination coverage, reduce under-five mortality and be cost-effective in terms of averted DALYs.

**Trial registration:**

This trial was retrospectively registered with ClinicalTrial.gov as NCT04913714 on the 4th of June 2021.

**Supplementary Information:**

The online version contains supplementary material available at https://doi.org/10.1186/s12889-025-25441-x.

## Introduction

Reducing under-five mortality (U5M) is a global call for action [1, 2]. While mounting evidence shows a declining global rate of under-five mortality, the rate remains high in low-and-middle-income countries (LMICs), especially in sub-Saharan Africa (SSA) at 74 deaths per 1000 live births compared to the global average of about 37 per 1000 live births [3, 4]. As the world aims for a global health convergence, each country is responsible for initiating interventions to reduce U5M progressively. Scientific evidence shows that about 5 million children worldwide died from preventable causes in 2020 before their fifth birthday and the rate of death was fourteen times higher in SSA [4]. Vaccine-preventable diseases such as measles, pneumonia, diarrhoea, and meningitis account for about 40% of U5M [5–8].

It is estimated that for countries in the World Health Organization (WHO) African region to meet the U5M target of 25 per 1000 live births, interventions must cause 70% reduction in U5M [4]. Some studies suggest mass radio campaigns to improve childhood immunization coverage may be a cost-effective way to dramatically reduce U5M [9]. This reduction may occur through changes in the health-seeking behaviour of parents, especially expectant mothers meeting the minimum recommended four visits of antenatal care and ensuring that each infant or child under five years is fully vaccinated against preventable child-killer diseases [10–13]. For example, one study in Burkina Faso projected that implementation of a radio campaign message at scale has the potential to increase health-seeking behaviours of pregnant women and mothers of under-five children, leading to an estimated 13,400 avoided U5M (lives saved) and close to four thousand averted disability-adjusted life years (DALYs) [14] among post-neonatal under-five children. Regardless of the potential positive impact of mass radio campaigns, there is paucity of studies evaluating its cost-effectiveness or cost-consequence [15]. While the former involves measuring costs against one health outcome, the latter requires targeting more than one health outcome [16, 17].

With support from multiple stakeholders, we initiated a three-month trial study involving a radio vaccination campaign program targeted at improving infant vaccination coverage and other outcomes such as antenatal visits and reported disease symptoms in two districts in Ethiopia (See subsequent sections for description of the trial). Evidence from the trial was convincing to enable a scale-up [18]. However, given resource constraint that is typical of most, if not all, low-income countries and the need for context-driven scientific evidence to stimulate government investment to scale up interventions, we decided to evaluate the potential impact of the trial at scale in Ethiopia.

Ethiopia, a low-income country in East Africa is the second most populated country in Africa and an interesting place to study for several reasons. For demographic reasons, the country is the second most populated country in Africa and had about 120 million inhabitants in 2021 of which up to 54% were women and 16% (19.2 million) were children under-five [19, 20]. For health reasons, the country accounted for about 65% of the world’s total episodes of severe pneumonia in 2011 and was ranked the fifth highest in terms of global mortality attributable to pneumonia and diarrhoea in 2013, of which children were the most victims [21]. As of 2021, the fertility rate in Ethiopia was 45.2% more than the world’s average of 2.3 per woman [19, 20, 22]. Again, infant and under-five mortality were 17.5% and 19.1% more than the world’s average [23]. Out-of-pocket health expenditure accounts for 27% of Ethiopia’s total health expenditure and about 5.4 million Ethiopians are exposed to catastrophic health spending [19, 24, 25]. Evidence from 2023 WHO data shows only 34% of under-five children were fully vaccinated based on confirmed vaccination cards in Ethiopia [25]. For reasons related to this study, household radio penetration/ownership in 2021 was 57.3% in large towns, 52.6% in small towns, and 34.8% in rural areas. Recent study shows that radio penetration in Ethiopia is significantly higher than television [26] which makes radio a preferred media for mass vaccination campaign to promote public health behaviour change.

In Sub-Saharan Africa, barriers to childhood immunization include lack of information or knowledge about vaccines [23, 27–29], unawareness of the need and value of immunization, forgetfulness [30, 31], vaccine misconceptions and low belief in vaccination [32–35], thus making a communication intervention key to addressing these barriers. Drawing on existing knowledge and using evidence from our trial study, we hypothesized that a well-designed and implemented mass radio vaccination campaign program targeted at health-seeking behaviour change such as childhood immunization may address multiple health outcomes not limited to only infants but all under-five children [36].

Deductively, this study answers three questions; (1) will the trial intervention be cost-effective by improving full vaccination coverage at scale in 18 radio community radio stations in Ethiopia, (2) how many lives will be saved in under-five children (U5M avoided), and (3) what will be the associated cost per DALY avoided?

## Methods

### Setting and intervention description

The intervention design was a prospective quasi-experimental trial involving a 10-minute vaccination radio drama, a 10-minute community health worker discussion on the radio, and a 30-minute phone-in by listeners. We named the intervention “10 + 10 + 30” such that the radio campaign program is used to promote public awareness to improve full vaccination coverage among infants in Ethiopia. See Table S1 in the supplementary file for titles of the episodes of the serial drama. Two districts in the Jimma Zone were selected for the trial. They were Manna district (intervention district) and Chora Botor district (control district). To the best of our knowledge, no other mass media campaign on childhood vaccination took place concurrently in the trial districts. As established elsewhere [10, 14, 37], the underlying theory of change regarding the intervention was that dramatization of educational campaigns using radio broadcasting has the potential to change behaviours and attitudes, whereby parents, especially mothers will be encouraged to fully vaccinate their children to avert disability-adjusted life years (DALYs). To commence the intervention, we organized an inception workshop to identify measurable outcomes and create precise message content for airing on Jimma Community Radio for three months. The workshop had eighteen participants comprising community health workers and officials from UNICEF Ethiopia, Jimma University, Ethiopia Broadcasting Corporation, and the Federal Ministry of Health. Besides the workshop, we organized a focused group discussion among mothers with infants and health extension workers to elicit perspectives on the opportunities and challenges of using a mass radio campaign to promote childhood vaccination in Ethiopia. The 3-month implementation of the intervention commenced on 23rd October 2020 and ended on 10th January 2021. Detailed presentations of trial development, registration, and implementation are publicly available at ClinicalTrial.gov *and* elsewhere [18], including seeking ethics approvals and informed consent.

### Costing approaches

Using a micro-costing approach, we valued and quantified the incremental economic and financial costs of the intervention compared with a no-intervention scenario (business as usual practice without mass radio vaccination campaign). Costs attributable to the intervention considered the opportunity costs of all relevant items/resources consumed during the trial, including those related to inception training workshops necessary to design and validate content used for the intervention. Consistent with others [14], the total cost of the intervention excluded research-related expenditures. Additional costs to providers due to the procurement of vaccines, immunization, and increased care-seeking were quantified and adjusted using unit cost information from other studies in the same setting [38, 39].

### Outcomes and perspective

Study outcomes were the proportion of incremental fully vaccinated under-five coverage, the associated lives saved (mortality avoided), and costs per averted DALY. We reported outcomes from the provider perspective and defined the provider as the government providing equal access to health services in Ethiopia.

### Estimating resources and costs

We evaluated intervention costs as the aggregated financial and economic costs of implementing the intervention. Campaign costs during the trial were used to project the total expenses at scale for 18 community radio stations because we believed that such a number of radio stations could help adequately reach rural dwellers in at least six regions. Cost components include the expenses of the inception workshop, radio drama design workshop, community health workers (CHWs) training workshop, cost of airtime, listener group mobilization through telephone calls, and the expenses on program implementation, coordination, and management costs. The provider costs buildup considered all expenses related to procurement, storage, deployment/immunization, and the opportunity costs of staff time allocated to immunization outreach/services. It also included other medical and non-medical service utilization costs derived from published sources that used data from Ethiopia [21, 38, 40, 41]. All costs were adjusted in 2021 purchasing power parity in international United States Dollars using a recommended web-based PPP converter [42, 43]. See Table S2 in the supplementary file for baseline intervention cost.

### Decision modelling

We developed a decision tree model that compares participants in the control and intervention groups based on the hypothesis that exposure to the intervention will increase coverage of fully vaccinated under-five children, consequently increasing care-seeking for under-five children. For the control group, we assumed a lesser coverage of fully vaccinated infants may occur due to limited knowledge about the health implications of incomplete vaccination. The model did not consider the costs of mortality as a consequential secondary outcome such as deaths due to vaccine preventable diseases. Model parameters were derived from the trial study [18] and complemented by provider cost parameters from secondary sources (See table S3 in the supplementary file).

### Measurement of effectiveness

During the trial, we determined vaccination coverage as the proportion of infants vaccinated between the two groups. In this study, we measured incremental effect as the outcome difference between the intervention arm and the control. The measurement considered post-neonatal under-five fully vaccination coverage and DALYs averted (primary outcomes) and the attributable lives saved, or mortality avoided (secondary outcome). Because the trial did not measure the effect of the intervention on under-five mortality, we adopted a scenario approach and used the recommended Lives Saved Tool (LiST) [9, 37] to model a 31.9% impact of the intervention in reducing under-five mortality in Ethiopia. We quantified discounted life years saved per child under-five using Ethiopia’s average life expectancy at ages 2.5-65 years in 2021, and discounted at 3% to about 30 years [5, 14, 19]. This study equates the estimated discounted life years saved with the intervention to discounted disability-adjusted life years (DALYs). Again, we quantified program costs per child under-five by dividing the total program costs by the total number of under-five children in Ethiopia. The same approach was used to estimate the provider costs per fully vaccinated child under-five years. Incremental cost-effectiveness ratio (ICER) was measured as the ratio of incremental provider costs to incremental DALYs averted among under-five children to determine the cost-effectiveness of the intervention. Outcome measurement and reporting followed the Consolidated Health Economic Evaluation Reporting Standard (CHEERS) checklist [44].

### Considerations in scale-up scenario

The trial focused on infants, while this current study assumed the potential benefit of mass radio campaigns for childhood vaccination extends beyond infants. Thus, this current study considered all under five children in Ethiopia in 2021 (Table S4). Outcomes during the trial included full vaccination coverage defined as infants who received all doses of Penta, Rota, pneumococcal conjugate vaccine (PVC), and oral poliovirus (OPV) vaccines as originally assessed in the baseline study. We also assumed that once the intervention aims for saturation of the vaccination campaign, it will encourage parents to ensure their children receive all other doses in the vaccine schedule, including Bacille Calmette-Guérin (BCG), measles-rubella, inactivated polio, and meningococcal A conjugate vaccines. These assumptions were incorporated into the lifesaving model to account for the distributional effects that may be attributable to each vaccine based on their effect size. The design of the intervention followed a need assessment to address the communication gap identified by the Ethiopia Extended Program for Immunization (EPI). It also aimed to involve health extension workers in community health education campaigns to improve childhood vaccination coverage and care-seeking for under-five children more generally. At scale, we considered full vaccination coverage as one of two primary outcomes because it provides immunity against major post-neonatal under-five killer diseases, including pneumonia, tuberculosis, meningitis, diarrhoea and measles [5, 25]. Using data from the trial and child mortality data from the WHO Sustainable Development Goal monitoring report [5] and other sources [45], including expert opinion, we predicted the impact of the intervention on study outcomes, considering national-level indicators and vaccination service utilization data obtained from Ethiopia Ministry of Health [46]. Despite projecting a 135% increase in full childhood vaccination coverage, equivalent to an estimated incremental 8,006,400 fully vaccinated under-five children, about 68.1% of under-five mortality in Ethiopia are caused by diseases not directly triggered by incomplete vaccination [45] Thus, we projected a 31.9% reduction in vaccine-attributable under-five mortality linked to pneumonia, tuberculosis, meningitis, diarrhoea, and measles. By the approach used, we assumed that 68.1% of under-five mortality may still occur with the intervention in place, leading to only 288,000 lives saved relative to a no intervention scenario.

Again, we considered the impact of household radio penetration and listening behaviours in Ethiopia as it is a means to delivering the intervention at scale and getting mothers to seek healthcare before and after childbirth to protect the health of their under-five children.

By focusing on the provider perspective, scale-up cost scenarios assumed each of the estimated 67 community radio stations required to broadcast the intervention nationwide will charge an equal amount corresponding to 21.2% of the total campaign cost. We assumed the provider (the government) will assign community health workers to assist with the intervention amounting to 25% of their working time. The unsubsidized unit cost of vaccination assumes the provider paid the full cost to procure the vaccines.

### Sensitivity analysis

We performed deterministic sensitivity analysis by subjecting the estimated ICER to one-way sensitivity analyses, which involved varying the 95% uncertainty intervals around the mean values of four input parameters. The varied parameters include the unit cost of the radio drama program (intervention) per child under-five years, the provider costs due to increased medical consultation (care-seeking for under-five children), the unsubsidized costs of full vaccination, and the estimated number of lives saved with the intervention. A willingness to pay threshold value for determining the cost-effectiveness of the intervention considered the proposed WHO-choice for evaluating country-specific cost-effectiveness of an intervention. Thus, an intervention is cost-effective if the estimated ICER is less than Ethiopia’s current gross domestic product (GDP) per capita of US$2559 in purchasing power parity equivalent in international US dollars in the program year 2021 [47]. In terms of DALY, an alternate decision rule, according to the WHO, is that an intervention costing less than US$100 is “good” and cost-effective while those that cost below US$25 are “excellent” [47].

## Results

### Provider campaign (intervention) costs

From the provider perspective, the overall economic cost of the immunization campaign program for three months in Ethiopia amounts to US$ 659,820. About 53% of the program cost was due to setup expenditures on workshops including inception, radio drama design, and training of community health workers. Costs of airtime (broadcasting of the intervention) correspond to 23.6% of the program costs. The estimated program cost per intended child under five years amounts to US$0.04 in economic cost and US$0.03 in financial cost. By adding the unit provider costs of full vaccination and increased consultation, the costs per child under-five years equals US$37.83 and US$36.33 in economic and financial costs, respectively (Table 1).

**Table 1:** Distribution of provider costs by activity over study duration

Immunization message campaign activity	Estimated financial costs at scale for 18 community radio stations countrywide. (2021 US$)	Estimated economic costs at scale for 18 community radio stations countrywide. (2021 US$)
Inception workshop	100 500	100 500
CHWs Training workshop	201 000	201 000
Drama design Workshop	13 950	13 950
^∞^Drama production	32 550	32 550
Airtime (broadcasting/airing of intervention)	155 520	155 520
Listener group mobilization (telephone calls)	27 000	27 000
Managing & coordinating the program in Ethiopia	100 500	100 500
Campaign support from CHWs and Experts (25% effort)	28 800	146,880
Overall campaign message costs	659 820	777 900
Total number of under-five children, 2021	19 200 000	19 200 000
Campaign cost per child < 5yrs	0.03	0.04
Provider costs attributable to vaccination and increase care-seeking for children under five years in Ethiopia		
Unsubsidized unit cost per vaccine dose	3.15 [2.60–3.70]	3.15 [2.60–3.70]
Unsubsidized costs of full vaccination	15.00 [13,–17]	15.00 [13,–17]
Provider costs per child due to increased consultation, excluding vaccination and campaign-related costs.	23.7 [16.1–31.3]	25.19 [21.8–28.6]
Provider costs per child < 5yrs	36.33	37.83
Incremental under-five children fully vaccinated	8 006 400	8 006 400
*Incremental provider costs over study period	290 907 465	302 886 240

*CHWs* Community Health Workers

*Multiply provider costs per child < 5yrs by incremental number of under five children fully vaccinated due to the intervention

∞Production in five languages namely Afar, Amharic, Oromo, Somali and Tigrinya

### Incremental effects attributable to the intervention

During the trial, the intervention resulted in a 134.7% change in full vaccination coverage among under-five children compared to a no-intervention scenario. At scale, the increase in vaccination coverage was projected to cause an estimated incremental 8,006,400 under-five children receiving full vaccine doses and an estimated 288,000 lives saved or mortalities avoided. Multiplying the projected lives saved with the intervention by the estimated discounted disability-adjusted life years of approximately 30 years per child yielded 8,735,040 total life years saved and DALYs averted (Table 2).

**Table 2:** Incremental effects attributable to the intervention at scale

Description	Without intervention *N* = 19,200,000	With intervention *N* = 19,200,000	Incremental effect (% change)	*p*-value
	*N* (%)	*N* (%)		
Fully vaccinated coverage at scale	5,625,600 (29.3)	13,632,000 (71.0)	8,006,400 (134.7)	< 0.001^∞^
Estimated mortalities in < 5 children	902,400 (16.0)	614,400 (4.5)	−288,000* (31.9)	-
Discounted life years	30.33	30.33		-
DALYs	27,369,792	18,634,752	−8,735,040**	-

*Mortality avoided equal lives saved

**Disability-Adjusted Life Years (DALYs) averted

^∞^Estimate based on trial data

### Cost-consequence results

The projected incremental annual economic and financial provider costs yielded about US$678 and US$615 million, respectively. About 27% of the projected additional costs were attributable to immunization service charges while 73% were associated with increased hospital resources used, that is, the direct and indirect costs of care-seeking excluding vaccination and campaign-related costs. Dividing the projected incremental provider costs by the estimated total DALYs averted generated about US$35 per DALY averted with the intervention (Table 3).

**Table 3:** Cost-effectiveness results attributable to the intervention

	Estimated financial cost-consequence from provider perspective (95% UI)	Estimated economic cost-consequence from provider perspective (95% UI)
Estimated number of lives saved in < 5 children (mortality avoided, 14.9% relative to no intervention scenario)	288,000 [215, 600]	288,000 [215,200–353,600]
Estimated life years saved*	8,735,040	8,735,040
Estimated DALYs averted	8,735,040	8,735,040
Estimated incremental provider costs	290,907,465	302,886,240
Estimated ICER	33.3	34.7

Multiply lives saved by the discounted 30.33 life years per beneficiary

*DALYs *Disability-Adjusted Life Years, *ICER* Incremental Cost-effectiveness Ratio

### Sensitivity results

**Fig. 1:**
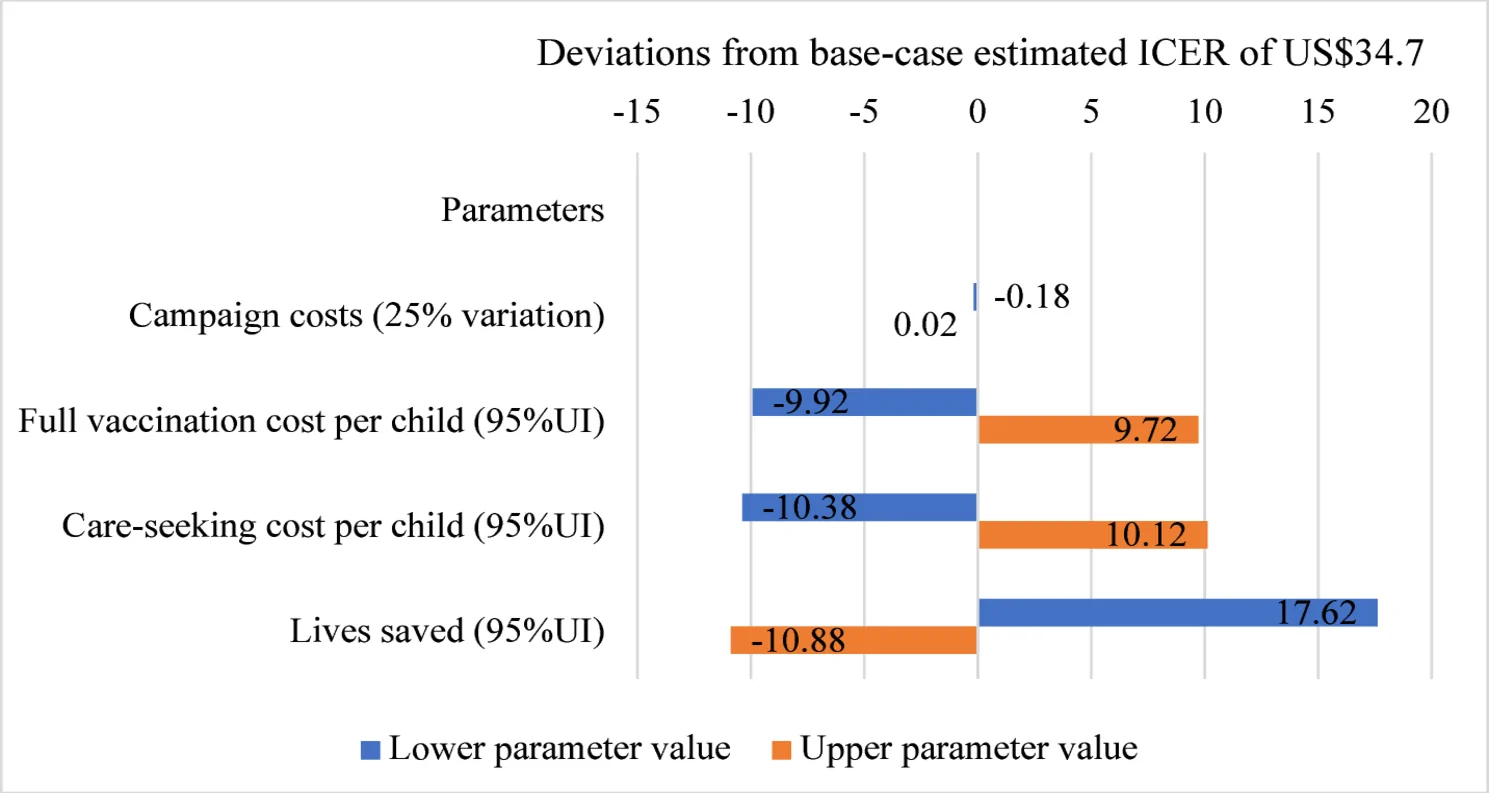
Tornado graph showing deviations from the base-case estimated ICER in a one-way deterministic sensitivity analysis using key uncertainty parameter variables

In descending order of magnitude, the results from the one-way deterministic sensitivity analysis show that the base-case estimated ICER was most sensitive to the estimated lives saved followed by the care-seeking cost per child < 5 years and full vaccination cost if the 95% uncertainty parameter values change one at a time. The radio campaign costs had the least impact on the estimated ICER (Fig. 1).

## Discussion

We used data from a trial [18] and other relevant sources to perform a cost-effectiveness evaluation to study the impact of a mass radio campaign program on childhood vaccination coverage at scale in Ethiopia and the associated potential lives saved and cost per DALY averted from a provider perspective. Relative to a no-intervention scenario, the intervention showed potential to increase full vaccination coverage by over 100%, accounting for an extra 8 million post-neonatal under-five children receiving all doses of vaccines before their fifth birthday. Again, had the intervention been implemented at scale, under-five mortality in Ethiopia would have declined by 31.9%, equivalent to about 288,000 lives saved. The projected reduction in under-five mortality or lives protected may occur because the campaign will increase and intensify awareness to cause more parents to seek care and vaccinate their children against potential child-killer diseases like pneumonia, tuberculosis, meningitis, diarrhoea and measles. As we observed during the trial, reported cases of diarrhoea, fever, cough, and difficulty breathing among beneficiaries in the intervention arm declined by 82.9%, 32.1%, and 41.5% relative to subjects in the control arm, respectively [18]. The margin of reduction was statistically significant, indicating that mass radio campaign has the potential to reduce under-five morbidity and, by extension, deaths.

Using Ethiopia’s GDP per capita of US$2,559 as a willingness to pay threshold, the estimated cost per DALY averted of about US$35 at scale shows that the intervention is cost-effective. Again, some studies [15, 47] note that interventions costing below US$100 per DALY are commendable and those less than US$20 per DALY are excellent, suggesting that our intervention is commendable. Moreso, as providers tend to spend more resources than patients pay for, we argue that the estimated cost per DALY avoided from the provider perspective would have been twice less had we estimated the outcomes from the patient or household perspective. The sensitivity results showed that the cost per averted DALY is about 18% and 17% sensitive to provider costs attributable to increased care-seeking for under-five children and the estimated number of lives saved (avoided mortality), respectively.

We argue that depending on the study perspective and setting, outcomes may vary. For example, using the LiST modeling approach, Head and colleagues [15] estimated a 16% to 23% reduction in child mortality (lives saved) in Burkina Faso, while in the same setting, others [9, 14] estimated between 5.5% and 7% annual reduction in child mortality. The uncertainties in reported outcomes may be attributable to considerations in the modeling. In this present study, we relied on several sources for data [4, 5, 45, 48], including expert opinion, to model the potential impact of the intervention on under-five mortality in Ethiopia. We considered several measures to minimize confounding factors. For instance, we checked vaccination cards for all targeted beneficiaries during the trial rather than relying on recall for proof of vaccination. Again, the trial design helped to measure outcomes concurrently for participants in the control and intervention districts and used the same considerations for the scale-up scenario. The campaign discussed a wide range of disease symptoms, permitting phone-ins by mothers wanting to know more about the benefits and dangers associated with complete and incomplete childhood vaccination, respectively. As reported in another study, we believe our study outcomes may indirectly be influenced by the complementarity effect of improved health seeking behaviours and other interventions such as those related to childhood nutrition supplementation [36, 49, 50]. Additionally, our model scenario considered that vaccination service provision in Ethiopia will have to increase three to four-fold to achieve the desired outcome. Currently, up to 868 health facilities across the country provide routine vaccination services, of which about 86.4% serve the rural population [46].

We assumed each of the estimated 18 radio stations and their broadcasting spots in communities and households served about 1.8 million people. However, we anticipate a cross-boundary effect of the campaign message may occur if some radio stations have frequency bandwidths exceeding others. As we know, variations in population density across regions and zones exist in Ethiopia, translating into differences in cost allocation to radio stations. That notwithstanding, we assumed the provider (government) has the ultimate bargaining power to pay each radio station the same amount, equivalent to about 21% of the total radio vaccination campaign costs.

While the design of this study is not new [13], the setting and approach contribute significantly to knowledge and literature as there is scant published studies on the cost-effectiveness of the radio vaccination campaign program in LMICs [47]. Existing studies in the same setting only reported costs of disease-specific response outcome such as cholera [40] and measles [38] while one recent study presented cost-effective estimate for a childhood pneumococcal vaccination program [21].

### Strengths and limitations

The strength of this study is closely linked to the feasibility, scalability and sustainability. For instance, we have shown that the low unit cost of the intervention is financially feasible in a low-income context like Ethiopia. Moreover, the design involved community health workers and local radio stations, making it feasible to leverage existing infrastructure at low cost to the government. Additionally, the estimated cost per DALY averted was about US$35, which is below the WHO threshold for cost-effectiveness, suggesting that scaling the intervention could be economically viable and attractive for policymakers. Also, the campaign framework can be adapted for national implementation, considering the moderate to high prevalence of radio penetration in Ethiopia. Nevertheless, sustainability of the intervention will depend on continued funding commitment in health communication strategies.

Regarding limitations with methods and design, a cost-effectiveness analysis approach limited our ability to evaluate the broader consequence or outcome of each vaccination as vaccine preventable diseases differ in severity and mortality risks. Also, the assumption that full vaccination may avert years lived with disability among under-five children may contribute to underestimation of the DALY. However, we remain optimistic in our evaluation as previously reported elsewhere [9, 14]. Additionally, scaling up the potential impact of the intervention for the whole of Ethiopia means that we assumed homogeneity of the study population. However, Ethiopia has diverse population and cultures that may affect vaccination care-seeking behaviours and outcomes. We believe such diversity be considered when interpreting results of this study. Moreso, there is high potential that COVID-19 lockdown restrictions impacted vaccination access and care-seeking behaviours in Ethiopia during the intervention study, and this may affect our outcome measures.

We argue that using some costs data obtained from secondary sources may have contributed to underestimation or overestimation of endpoint costs. However, the approach used is standard practice in economic evaluation [16]. For the intervention to yield the estimated outcomes, the scale-up scenario assumed a 100% radio penetration but was about 50% [22]. This means that more investment will be required to expand radio infrastructure to make the intervention accessible to all priority beneficiaries. It is also possible that mothers can leverage mobile phone technology to access health promotion information via radio broadcast.

## Conclusion

The 10 + 10 + 30 radio campaign on childhood vaccination was a cost-effective intervention regarding increasing full vaccination coverage and averted DALYs of under-five mortality in the intervention district in Ethiopia. The study provides evidence to show that a radio vaccination campaign at scale in Ethiopia may have the potential to increase full childhood vaccination coverage, reduce under-five mortality and be cost-effective in terms of averted DALYs. Nevertheless, because the study outcomes are based on modelled estimates, the conclusions should be interpreted with caution.

## Supplementary Information

Supplementary Material 1.

## Data Availability

The dataset supporting the conclusions of this article is included within the article.

## Abbreviations

DALYs: Disability-Adjusted Life Years
LMICs: Low- and middle-income countries
GDP: Gross Domestic Product
U5M: Under-five mortality
SSA: sub-Saharan Africa
WHO: World Health Organization
ICER: Incremental cost-effectiveness ratio
