# Supplementary material for: Cost-effectiveness evaluation of a radio campaign to improve full vaccination coverage among children under-five in Ethiopia
Source: BMC Public Health. 2026 Jun 16;26:2681. doi: 10.1186/s12889-025-25441-x (PMC13613585; doi:10.1186/s12889-025-25441-x)
Supplement: Supplementary file 1 [file 12889_2025_25441_MOESM1_ESM.docx]

**Supplementary file for the manuscript: “Cost-effectiveness evaluation of a radio campaign to improve full vaccination coverage among children under-five in Ethiopia”.**

**Table S1** Radio drama episodes on childhood vaccination in Jimma, Ethiopia

| Week | Drama Episode/Topic |
| --- | --- |
| 1 | INTRODUCTION |
| 2 | POLIO VACCINATION |
| 3 | TUBERCULOSIS VACCINATION |
| 4 | DIPHTHERIA, TETANUS, AND PERTUSSIS VACCINATION |
| 5 | HEPATITIS (HEP-B) AND HAEMOPHILUS INFLUENZA-B (HIB) VACCINATION |
| 6 | DIARRHEA AND PNEUMONIA |
| 7 | MEASLES AND MEASLES VACCINATION |
| 8 | ADVANTAGES OF CHILDHOOD VACCINATIONS & AVOIDING DROPOUT |
| 9 | ROLES AND RESPONSIBILITIES OF PARENTS, COMMUNITY LEADERS & HEALTH DEVELOPMENT ARMY |
| 10 | ROLES AND RESPONSIBILITIES OF HEALTH PROFESSIONALS AND HEALTH EXTENSION WORKERS |
| 11 | GOVERNMENT PROGRAM ON VACCINATION |
| 12 | CONCLUSION |

Lives saved, DALY averted and costs.

Fully vaccinated and increased hospital resource use

Intervention

Non-fully vaccinated coverage

Study group

Lives saved, DALY averted and costs.

Fully vaccinated and hospital resource use

Control

Non-fully vaccinated coverage

**Figure S1**. Decision tree model for evaluating the cost-effectiveness of radio vaccination program targeted at under-five children in Ethiopia.

Table S2. Intervention costs at baseline

|  | Budget Description/category | Total budget amount agreed in USD |
| --- | --- | --- |
| 1 | Inception Workshop | 1,500.00 |
| 2 | Radio Drama design Workshop | 7,200.00 |
| 3 | CHWs Training workshop | 3,000.00 |
| 4 | Drama design Workshop (Including scriptwriter production) | 9,600.00 |
| 5 | Data collection fieldwork | 1,500.00 |
| 6 | Air time (24*@ 60 min each @$ 3/min | 8,640.00 |
| 7 | Listener group mobilization (telephone calls) | 1,500.00 |
| 8 | Managing & Coordinating program in Ethiopia | 1,500.00 |
| 9 | 2 Co-PIs in Ethiopia (30% efforts) | 9,180.00 |
|  | Sub Total | 43,620.00 |

Table S3. Decision model parameters

|  | **Parameter value**  **[95% UI]** | **Source** |
| --- | --- | --- |
| Probability of fully vaccinated children<5yrs due to intervention. | 0.710 | Baseline study [18] |
| Probability of fully vaccinated children<5yrs without intervention. | 0.293 | Baseline study [18] |
| Probability of disease exposure with intervention | 0.102  [0.07 - 0.135] | Baseline study [18] |
| Probability of disease exposure without intervention | 0.349  [0.297 - 0.401] | Baseline study [18] |
| *Adjusted unsubsidized unit cost per full vaccine doze (Some vaccines require more than one doze) | 5.75  [2.80 – 6.90] | [21,38,40,41,48] |
| *Adjusted unsubsidized costs of full immunization. | 23.00  [18 – 28] | [21,38,40,41,48] |
| *Adjusted provider costs excluding vaccination and campaign costs. | 61.5  [46.1 – 76.9] | [21,38,40,41,48] |

*Cost is adjusted to 2021 Purchasing Power Parity (PPP) in international US dollars.

Table S4. Indicators for national scale-up

| **Population** | **Ethiopia** | **Source** |
| --- | --- | --- |
| Total population, 2021 (million) | 120 | Ethiopia Statistics Service [22] |
| Women population (million) | 53.8 | Ethiopia Statistics Service, [22] |
| Fertility rate (birth per woman) 2023 | 4 | UNFPA [20] |
| Under 5 children, 16% of total population (million) | 19.2 | UNICEF Ethiopia, 2018 |
| . |  |  |
| **Health** |  |  |
| Crude birth per 1000 people, 2021 | 32 | World Bank (WDI) [19] |
| Infant mortality per 1000 live birth, 2021 | 39 | World Bank (WDI) [19] |
| Under-5 mortality per 1000 live births, 2021 | 46.8 | World Bank (WDI) [19] |
| Life expectancy at birth, 2021 | 65 | World Bank (WDI) [19] |
| Total number of health facilities providing routine vaccination services^∞^. | 868 | Ethiopia Ministry of Health [46]. |
|  |  |  |
| **Radio penetration*** |  |  |
| Radio ownership, National (%), 2016 | 30.5 | Ethiopia Statistics Service [25] |
| Radio ownership in rural areas (%), 2016 | 24.8 | Ethiopia Statistics Service [25] |
| Radio ownership in small towns (%), 2016 | 42.6 | Ethiopia Statistics Service [25] |
| Radio ownership in large towns (%), 2016 | 47.3 | Ethiopia Statistics Service [25] |
|  |  |  |
| **Economic** |  |  |
| GDP per capita, 2022 (Current, USD) | 1,028 | World Bank (WDI) [19] |
| CHE, % of population | 4.9 | Barasa et al. [23] |
| OOP as % Total Health Expenditure, 2021 | 27 | Barasa et al. [23] |

^∞^Other hospital resource used considerations included number of consultations per beneficiary and staff distribution.

*We assumed a 10% to 20% upward adjustment between 2016 to 2021 based on expert opinion backed by the growing penetration of technological advancement.

CHE – catastrophic health expenditure, OOP – out-of-pocket expenditure
